# Detection and Enumeration of Spore-Forming Bacteria in Powdered Dairy Products

**DOI:** 10.3389/fmicb.2017.00109

**Published:** 2017-01-31

**Authors:** Aoife J. McHugh, Conor Feehily, Colin Hill, Paul D. Cotter

**Affiliations:** ^1^Food Bioscience Department, Teagasc Food Research CentreCork, Ireland; ^2^School of Microbiology, University College CorkCork, Ireland; ^3^APC Microbiome InstituteCork, Ireland

**Keywords:** next generation sequencing, spore-forming bacteria, dairy, dairy powder, pathogens

## Abstract

With the abolition of milk quotas in the European Union in 2015, several member states including Ireland, Luxembourg, and Belgium have seen year on year bi-monthly milk deliveries to dairies increase by up to 35%. Milk production has also increased outside of Europe in the past number of years. Unsurprisingly, there has been a corresponding increased focus on the production of dried milk products for improved shelf life. These powders are used in a wide variety of products, including confectionery, infant formula, sports dietary supplements and supplements for health recovery. To ensure quality and safety standards in the dairy sector, strict controls are in place with respect to the acceptable quantity and species of microorganisms present in these products. A particular emphasis on spore-forming bacteria is necessary due to their inherent ability to survive extreme processing conditions. Traditional microbiological detection methods used in industry have limitations in terms of time, efficiency, accuracy, and sensitivity. The following review will explore the common spore-forming bacterial contaminants of milk powders, will review the guidelines with respect to the acceptable limits of these microorganisms and will provide an insight into recent advances in methods for detecting these microbes. The various advantages and limitations with respect to the application of these diagnostics approaches for dairy food will be provided. It is anticipated that the optimization and application of these methods in appropriate ways can ensure that the enhanced pressures associated with increased production will not result in any lessening of safety and quality standards.

## Introduction

**FIGURE 1:**
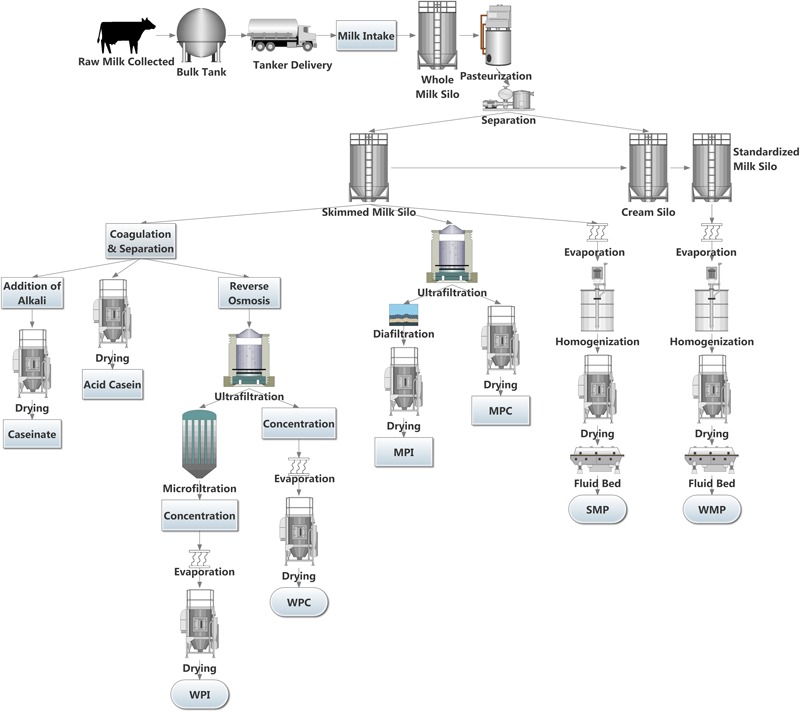
**Sample dairy powder production pipelines**.

The European Union’s removal of milk quotas in April, 2015 led to a 2% increase in milk deliveries to dairies in the EU for 2015. Some countries are taking full advantage of the new limitless system in the EU, with Ireland, Luxemburg, and Belgium increasing bi-monthly milk deliveries to dairies by in excess of 20% (Eurostat, 2016). Although the production rate has slowed in some other major dairy exporters, including New Zealand and Australia, the US has seen continued increases in production (Dairy Australia, 2015; DCANZ, 2016; USDA, 2016). The surplus milk produced can be processed into a wide variety of dairy products, including yogurt, butter, cheeses, and dairy powders. Dairy powders are a popular commodity due to their long shelf life, ease of storage and versatile nature. A wide variety of dairy powders can be produced, each with individual properties. These include whole milk powder (WMP), skimmed milk powder (SMP), whey protein concentrate (WPC), whey protein isolate (WPI), milk protein concentrate (MPC), milk protein isolate (MPI), casein and caseinates (Lagrange et al., 2015). Dairy powders can be used in fortification of other dairy products (Karam et al., 2013), as well as an ingredient in a wide array of foods including soups and sauces, confectionary (Sharma et al., 2012), infant formula, sports dietary supplements and in foods for health recovery (Gill et al., 2001; Lagrange et al., 2015). However, the increased production of dairy powders may create safety and economic risks to the dairy sector, specifically when controlling microbial loads in these products. Several key steps are involved in producing dairy powders including pasteurization, separation, evaporation, and spray drying (**Figure 1**). These thermal and mechanical processes can reduce the microbes present in the milk. However, spore forming bacteria may survive. It has been shown that the spore-forming bacterial composition of raw milk differs considerably from their associated dairy powders (Miller et al., 2015), highlighting that the processing of milk into powder changes the composition of the specific spore-formers present. Post-production, powders can be stored for extended periods and in the absence of water, bacterial metabolic activity and growth is limited (Deng et al., 2012), thus preventing spoilage and product defects. However, under these conditions, bacterial spores can remain dormant until more favorable conditions are encountered, when germination and outgrowth can proceed (Setlow, 2003, 2014).

## Bacterial Contaminants of Dairy Powders

### Sources of Bacterial Contamination of Dairy Powders

Spore-forming bacteria can contaminate dairy powders through a variety of means. Bacteria can originate from the soil (Heyndrickx, 2011), feces, bedding, feed, or milking equipment (Gleeson et al., 2013), or can enter the raw milk *via* contaminated teats, milking cups and bulk tanks. Additionally, contamination can occur during transport from the farm to the processing plant (Pantoja et al., 2011), and also within the processing facility itself from poor handling and contaminated equipment (Burgess et al., 2010; Faille et al., 2014). The formation of homogeneous or heterogeneous multicellular bacterial communities on the surface of processing equipment in the form of biofilms is a particular concern for the dairy processing sector and, when present, can lead to recurring problems of microbial contamination. The biofilms, which are themselves resistant to cleaning, can serve as a reservoir for bacterial spores which can slough off and contaminate dairy powders (Branda et al., 2001; Faille et al., 2014).

### Common Bacterial Contaminants

Common contaminants identified in dairy powders include species of the class Bacilli (**Table 1**), many of which are capable of forming endospores (Checinska et al., 2015). Taxa other than Bacilli have also been found to contaminate powdered dairy products with species reported including *Clostridium halophilum, Klebsiella oxytoca* (Buehner et al., 2015), *C. perfringens, C. septicum, C. novyi/haemolyticum, C. sporogenes* (Barash et al., 2010), *Staphylococcus aureus* (Zhang et al., 2015), and *Cronobacter sakazakii* (Minami et al., 2012). Bacteria of the genus *Clostridium*, as well as many of the contaminants of the class Bacilli (**Table 1**), including *Bacillus, Anoxybacillus, Geobacillus, Lysinibacillus, Brevibacillus*, and *Paenibacillus*, have a considerable advantage due to being capable of forming stress-resistant endospores. These genera, and their associated species, vary considerably with respect to the range of temperatures in which they can grow, and include some psychrophilic (Ivy et al., 2012) and thermophilic (Burgess et al., 2010; Watterson et al., 2014) species. Dairy product contaminating spore-formers can also differ by virtue of preferring anaerobic (Doyle et al., 2015) or aerobic (Gopal et al., 2015) conditions. Although many spore-formers are not pathogenic and are seen primarily as indicators of poor hygiene during milk collection and or processing (Burgess et al., 2010), some can cause disease (Andersson et al., 1995). Of the spore-formers identified in powders, specific representatives of *Clostridium* spp. and *Bacillus* spp. are the most worrying from a food safety point of view. *Clostridium* are anaerobic spore-formers, of which *C. botulinum* is the most notorious due to its highly potent botulinum toxin. There are many types of botulism including foodborne botulism, wound botulism, infant botulism and adult intestinal botulism. Infant botulism is the most common form (Sobel, 2005). Strains of *C. botulinum* isolated clinically have been identified in containers of opened milk powder from the home of patients with infant botulism (Brett et al., 2005; Johnson et al., 2005). Despite this, and although many species of *Clostridium* have been identified in dairy powders (Barash et al., 2010; Buehner et al., 2015), dairy powders have never been found to be responsible for a case of infant botulism (Brett et al., 2005; Johnson et al., 2005; Doyle et al., 2015). However, it is worth noting that anaerobic spore-forming bacteria, like *C. botulinum*, are less common than aerobic spore-formers in dairy powders. This may be due to the high degree of aeration involved in dairy powder processing or that testing criteria for spore-formers has been optimized to identify aerobic spore-formers except in the case of phenotype based assays for specific groups of anaerobic species. The ability of certain *Clostridium* species to reduce sulphite to sulfide under anaerobic conditions resulting in black colonies on specific media has been widely utilized. The accuracy of these qualitative and quantitative approaches has previously been discussed (Doyle et al., 2015). Of the aerobic spore-formers identified, the majority have been of the genus *Bacillus* (**Table 1**). Many species of this genus are generally regarded as safe and some are even used as probiotics (Hong et al., 2005); e.g., Bactisubtil, Biovicerin and Biosubtyl containing *B*. *cereus*, Bidisubtilis containing *B*. *subtilis*, Biosporin and Primal Defense containing *B*. *subtilis* and *B*. *licheniformis*, Biosubtyl containing *B*. *pumilus*, Enterogermina containing *B*. *clausii* and Lactospore containing *B*. *coagulans* (Hong et al., 2005). Other species of *Bacillus* have been used in the production of animal feed-stuffs; e.g., *B. subtilis* has been utilized for the fermentation of indigestible by-products of soya bean oil production to yield a suitable food source for monogastric animals (Wongputtisin et al., 2014). *B. cereus* sensu lato is the most important group of species identified from a pathogenic perspective (Bottone, 2010). This group, containing up to 11 individual, highly related species (Okstad and Kolsto, 2011; Liu et al., 2015), includes species that are regarded as non-pathogenic (Okstad and Kolsto, 2011). Other species include *B. thuringiensis* which is used as pesticides (Schnepf et al., 1998; Bravo et al., 2013); *B. cereus*, a class 2 pathogen capable of food poisoning which gave this species group its name (Bottone, 2010) and even a class 3 human pathogenic species, *B. anthracis* (Rasko et al., 2005). All of these are notoriously difficult to classify and differentiate from each other (Helgason et al., 2000; Radnedge et al., 2003; Rasko et al., 2005; Liu et al., 2015). *B. cereus* is the main cause of food poisoning from within this group. *B. cereus* strains can contain many enterotoxins which are associated with diarrheal food poisoning including non-hemolytic enterotoxin (Nhe; Lund and Granum, 1996; Lindback et al., 2004), hemolysin BL (Hbl; Beecher and Wong, 1997), and cytotoxin K (CytK; Lund et al., 2000). It should be noted that the description of CytK as a viable enterotoxin has been called into question as, in isolation, the presence of the corresponding gene has not been linked to virulence in diarrheal pathogenesis (Castiaux et al., 2015). Other molecules previously thought to be enterotoxins associated with food poisoning but which have since been reclassified include EntFM (Tran et al., 2010) and BcET (Choma and Granum, 2002). Some strains of *B. cereus* also produce an emetic toxin, cereulide (Ces), a product of non-ribosomal peptide synthesis, which can cause emetic food poisoning (Horwood et al., 2004; Toh et al., 2004).

**Table 1:** Contaminants of the class Bacilli identified in powdered dairy products.

Bacilli contaminants	Reference
*Bacillus lichenformis*	Ronimus et al., 2003; Ruckert et al., 2004; Rueckert et al., 2005; Reginensi et al., 2011; Buehner et al., 2015; Miller et al., 2015; Sadiq et al., 2016; VanderKelen et al., 2016
*Bacillus subtilis* sensu lato	Ronimus et al., 2003; Ruckert et al., 2004; Rueckert et al., 2005; Reginensi et al., 2011; Miller et al., 2015; Sadiq et al., 2016
*Bacillus pumilus*	Ruckert et al., 2004; Reginensi et al., 2011; Buehner et al., 2015; Miller et al., 2015; Sadiq et al., 2016; VanderKelen et al., 2016
*Bacillus circulans*	Ruckert et al., 2004; Sadiq et al., 2016
*Bacillus coagulans*	Ruckert et al., 2004; Sadiq et al., 2016
*Bacillus cereus* sensu lato	Reyes et al., 2007; Buehner et al., 2015; Miller et al., 2015; Sadiq et al., 2016; Zhang et al., 2016
*Bacillus megaterium*	Reginensi et al., 2011; Buehner et al., 2015
*Bacillus sonorensis*	Buehner et al., 2015; Sadiq et al., 2016
*Bacillus altitudinis*	Buehner et al., 2015
*Oceanobacillus* spp.	Buehner et al., 2015
*Bacillus clausii*	Miller et al., 2015; Sadiq et al., 2016
*Bacillus thermoamylovorans*	Miller et al., 2015; Sadiq et al., 2016
*Anoxybacillus* spp.	Miller et al., 2015; Trmcic et al., 2015; Sadiq et al., 2016
*Anoxybacillus flavithermus*	Ronimus et al., 2003; Ruckert et al., 2004; Rueckert et al., 2005; Reginensi et al., 2011; Sadiq et al., 2016; VanderKelen et al., 2016
*Geobacillus* spp.	Miller et al., 2015; Trmcic et al., 2015
*Geobacillus stearothermophilus*	Ronimus et al., 2003; Ruckert et al., 2004; Rueckert et al., 2005; Buehner et al., 2015; Sadiq et al., 2016
*Geobacillus thermoleovorans* group	Sadiq et al., 2016; VanderKelen et al., 2016
*Ureibacillus* spp.	Miller et al., 2015
*Urebacillus thermosphaericus*	Ruckert et al., 2004
*Aeribacillus pallidus*	Miller et al., 2015; Sadiq et al., 2016
*Lysinibacillus* spp.	Miller et al., 2015
*Lysinibacillus sphaericus*	Sadiq et al., 2016
*Paenibacillus* spp.	Miller et al., 2015
*Paenibacillus cookii*	Sadiq et al., 2016
*Paenibacillus macerans*	Sadiq et al., 2016
*Bacillus aerophilus* sensu lato	Sadiq et al., 2016
*Brevibacillus brevis*	Sadiq et al., 2016
*Brevibacillus parabrevis*	Sadiq et al., 2016
*Virgibacillus proomi*	Sadiq et al., 2016
*Bacillus shackletonii*	Sadiq et al., 2016
*Sporosarcina contaminans*	Sadiq et al., 2016
*Laceyella sacchari*	Sadiq et al., 2016
*Bacillus amyloliquefaciens*	VanderKelen et al., 2016

#### Spore Formation

Endospores are formed in *Bacillus* and *Clostridium* species in response to environmental stress, by the activation of the master transcriptional regulator Spo0A (Hoch, 1993) following a cascade of phosphorylation including five autokinases and two phosphorelay proteins (Molle et al., 2003). Spo0A binds to DNA and influences the expression of over 500 genes (Molle et al., 2003). It does so directly, for example it can control efficient replication of a single chromosome for both the mother cell and fore spore by binding to the origin of replication in the mother cell (Boonstra et al., 2013). But it can also work indirectly, through regulation of other transcription factors (Molle et al., 2003). There are over 100 genes known to be required for spore formation, with more being identified as research in the field develops (Meeske et al., 2016). Steps involved in spore formation include segregation of DNA, formation of a septum, engulfment and formation of a fore spore, formation of spore protein layers, cortex, membranes and spore coat and maturation of the spore before lysing the mother cell and being released. This process has previously been comprehensively reviewed elsewhere (Sella et al., 2014; Pompeo et al., 2016). Following its formation, an endospore can remain dormant and can persist in unfavorable environmental conditions without moisture or nutrients due to the protective structure and properties of the endospore.

#### Spore Structure

Endospores contain several thick layers. The outer coat, or exosporium, is a thick layer only found in some species, usually those of *B. cereus* sensu lato (Matz et al., 1970; Lai et al., 2003). The exosporium contains two layers, a basal layer surrounded by an external layer with hair like projections consisting mainly of the glycoprotein *Bacillus* collagen-like protein A (BclA; Sylvestre et al., 2002; Stewart, 2015). The exosporium, and especially BclA, contributes to hydrophobicity and aids the binding of spores to their substrates, including food preparation surfaces and stainless steel. This, along with its ability to assist spores in their avoidance of innate immune cells (Stewart, 2015), and also aids the spores’ survival, spread and pathogenicity potential in the food chain. The exosporium, if present, surrounds the spore coat. The spore coat is a complex, semipermeable, proteinaceous layer found on all endospores. It is the outermost layer of *B. subtilis* spores (Setlow, 2006) and gives resistance to chemicals and enzymes, as well as structurally holding the spore together. It excludes large molecules, while allowing nutrients pass through and interact with germination receptors deeper in the spore structure (Driks, 2002; Lai et al., 2003). The spore coat surrounds an outer membrane, which encapsulates the cortex. The cortex is made of specific peptidoglycan (Popham, 2002) that is assembled into rod shaped structures, located perpendicularly to the spore surface (Li et al., 2016). It confers resistance to wet heat and is essential in the dormancy of the spore as well as reducing the water content of the core (Setlow, 2006). The cortex surrounds the germ cell wall, which becomes the bacterial cell wall following germination (Setlow, 2006; Wells-Bennik et al., 2016). The germ cell wall surrounds an inner membrane. This too protects the bacterial spore against chemicals, and contains the proteins required for germination back to active cells (Setlow, 2003). Proteins including transporters (some of which are associated with eﬄux processes and unique to the spore inner membrane), proteases (essential for sporulation and germination), DNA repair and replication enzymes (including nucleotide excision repair enzymes, spore specific lyases and endonucleases), heat shock proteins and proteins involved in control of cellular processes in response to stress (including, but not limited to UV and oxidative stress) have all been identified in the spore inner membrane (Zheng et al., 2016). These all contribute to the resistance and persistence of spores in unfavorable conditions. Inside the inner membrane is the core of the endospore, which is severely dehydrated and compacted. This dehydration allows immobilization of proteins, preventing their coagulation following heat denaturation (Sunde et al., 2009). The core also contains high levels (up to 15–25% of the spores dry weight) of dipicolinic acid (DPA), most of which is chelated by divalent ions, allowing protection of spore DNA from external stressors as well as synthesis of new DNA in response to UV radiation (Setlow, 2006, 2007; Sunde et al., 2009). Also found in the spore core of *Bacillus* species is a group of small, acid-soluble spore proteins (SASP) of the α/β-type. These bind DNA in the spore core and alter its structure, thus aiding its resistance to heat, chemicals, UV radiation, and osmotic pressure (Setlow, 2006, 2007).

#### Survival of Spore-Forming Bacteria in Processing Environments

Spores can survive processing to which vegetative cells would normally succumb. Such processing-related stressed include desiccation, dry and wet heat, UV radiation, mechanical agitation, γ-radiation, chemical exposure and hydrostatic and osmotic pressure (Nicholson et al., 2000; Setlow, 2006). Indeed, while the temperatures and drying conditions used in the processing of milk to powders kills most vegetative bacterial cells, it also inadvertently selects for these spore-formers. Once powders are rehydrated, the spores may germinate by activation of germination receptors, either in response to nutrients called germinants (Setlow, 2003) or by heat activation (Luu et al., 2015). Germination independent of these receptors may also be triggered by calcium chelated dipicolinic acid (CaDPA), dodecylamine, or peptidoglycan fragments, although these mechanisms may not be applicable to the food industry (Setlow, 2014). Germination initiated by high pressure, either by activation of germination receptors or independent of them, can also occur (Setlow, 2014). Following germination, these spore-formers can proliferate in the absence of competition from other bacteria that were eradicated during processing (Brown, 2000).

## Legislation Governing Bacterial Contamination in Dairy Powders

Guidelines governing the levels and types of bacteria permitted in dairy powders are not very comprehensive, except in the case of infant formula. There are many different governing bodies that have set testing parameters; including the U.S. Food and Drug Administration (FDA), Food Standards Australia New Zealand (FSANZ), and The European Commission (EC). In Ireland, the Food Safety Authority of Ireland (FSAI) implements limits based on the Commission Regulation (EC) No 2073/2005 (European Commission, 2005). FSAI state that aerobic colony counts in dairy powders should ideally be <10^4^ cfu/g (FSAI, 2014). However, this is not a legal obligation, and does not mean that the food is unsafe as characterization of the species isolated would need to be performed in order to determine product safety. The U.S. Department of Agriculture (USDA) implements the following microbial limits in US extra grade dairy powders using the standard plate count; dry buttermilk <20,000 cfu/g (USDA, 2001a), dry whey <30,000 cfu/g (USDA, 2000), dry whole milk <10,000 cfu/g (USDA, 2001b), dry casein (acid) <30,000 cfu/g (USDA, 1968), instant non-fat dry milk <10,000 cfu/g (USDA, 2013), non-fat dry milk (roller dried) <50,000 cfu/g (USDA, 1984), and non-fat dry milk (spray process) <10,000 cfu/g (USDA, 2001c). The US Dairy Export Council (USDEC) implements limits for US dairy powders destined for international customers with limits on aerobic spore-formers set to between <500 cfu/g and <1000 cfu/g for thermophilic and mesophilic spores, respectively, in SMP, non-fat dry milk and WMP destined for infant powder, and <500 cfu/g and <2000 cfu/g, respectively, in SMP and WMP (Watterson et al., 2014).

In Australia and New Zealand, state agencies enforce limits set by FSANZ. *B. cereus* must be <100 cfu/g in 4/5 samples, and <1,000 cfu/g in 1/5 samples in dried milk powder and powdered infant formula products with added lactic acid producing cultures, and must be absent in five samples of 1 g in powdered infant formula. The EC regulation, as amended (European Commission, 2005) sets similar legal microbiological criteria including a limit of <50 cfu/g presumptive *B. cereus* in 4/5 samples and <500 cfu/g in 1/5 analyzed is set in accordance to EN/ISO 7932 (Standards, 2004).

Due to the competitive market for dairy ingredients, individual purchasers often set their own microbiological limits to ensure high standards. In many cases dairy powders will not receive any further treatments before incorporation into other products. For example, powdered infant formula manufacturers often have close relationships with the dairy powder supplier to ensure high microbiological standard are met, and set strict criteria (Kent et al., 2015).

## Detection of Spore-Forming Bacteria

Apart from dairy powder that is due for export from the US, no legislation thoroughly covers the enumeration or identification of all spore-formers in dairy powder. This is in spite of recent research highlighting the need for accurate spore quantification and identification (Burgess et al., 2010). Identification and enumeration of all spore-formers present in dairy powders allows identification of potential problematic species whether from a hygiene, quality or pathogenic perspective. This information would allow manufacturers implement more comprehensive and/or directed preventative measures (Pennacchia et al., 2014) resulting in continued economic and safety confidence in the sector. Understanding composition of total spore-formers within a product contributes to a clearer understanding of the source of potential quality or safety issues should they arise and allows faster implementation of control measures (Burgess et al., 2010; Pennacchia et al., 2014). Indeed, efforts have continued to be made in recent years to improve the detection and identification of spore-forming bacteria present in dairy powders (Watterson et al., 2014; Miller et al., 2015; Sadiq et al., 2016).

### Culture Based Methods

#### Spore Count Methods

Typical spore count tests involve the heating of a reconstituted powder sample to 80°C for 12 min before cooling, culturing and enumerating colonies (Frank and Yousef, 2004; Watterson et al., 2014). Highly thermo-resistant spores are selected by heating to 100°C for 30 min before cooling and culturing while numbers of especially thermo-resistant spores are quantified by heating to 106°C for 30 min, cooling and culturing. Media is incubated in the presence or absence of oxygen to select for aerobic or anaerobic spore-forming species, respectively. Incubation can also be at different temperatures. Incubation at 6°C will select for psychrophilic spore-formers, incubation at 30–35°C will select for mesophilic spore-formers and incubation at 55°C will select for thermophilic spore-formers (Watterson et al., 2014; Kent et al., 2016). Further analysis of isolated colonies is required in order to determine the species present, and the options available for this analysis are discussed at a later stage in this review (see Culture-Based Identification of Spore-Forming Species and Post-Culture DNA-Based Classification Methods). Total bacterial counts and spore counts, although informative, are not without their limitations. Almost a century ago it was highlighted that different media will result in different bacterial counts (Ayers and Mudge, 1920) and that more than just quantitative data is needed with respect to contamination of dairy products, in order to determine the significance of the contamination (Ayers and Mudge, 1920). The use of various heating methods is somewhat redundant in terms of identification of different species (Miller et al., 2015). However, the actual abundance of these spore-forming bacteria does differ depending on the test method used (Kent et al., 2016). In order to get a clear picture of the total spore-former composition present in a powder sample through culture-based approaches, a variety of incubation conditions, temperatures, agars and, possibly, heat treatments would be needed. This highlights the need for stronger/more robust test methods to determine the abundance of (spore-forming) bacteria in dairy powders.

#### Culture-Based Identification of Spore-Forming Species

Numerous culture-based tests have been developed in order to help identify spore-forming bacteria. These involve the use of selective media and, in some cases, additional tests to provide further information regarding the identity of the species present. Both Bacara and Mannitol Egg Yolk Polymyxin (MYP) agars have been developed for the isolation of *B. cereus.* The testing used for presumptive *B. cereus* in Europe (Standards, 2004) involves the use of MYP agar and the hemolysis test. However, MYP has been shown to be not as selective as Bacara agar for *B. cereus* (Tallent et al., 2012), potentially leading to false positives. Some *Clostridium* species, the sulphite reducing Clostridia (SRCs), have the ability to reduce sulphite to sulfide under anaerobic conditions. A number of sulphite containing agars have been developed for their selection (Wilson and Blair, 1924; Gibbs and Freame, 1965; Weenk et al., 1995). SRCs are identified by a black color change, however, other bacteria capable of reducing sulphite and can also grow on these media, these are referred to as sulphite reducing bacteria (SRBs) (Doyle et al., 2015). Other tests can involve analyzing phenotypes by visualizing morphological properties and performing biochemical tests to narrow down the possible species (Janda and Abbott, 2002; Reyes et al., 2007).

#### Limitations of Culture-Dependent Analysis

A common limitation with all of the aforementioned methods is a requirement that the bacteria first be cultured. This can result in important difficult-to-culture species being overlooked due to inappropriate culturing conditions, temperature, aeration, and/or media type. Furthermore, colony selection may favor the selection of the largest/most plentiful colonies above the smaller/less plentiful types. Although these methods allow isolation and enumeration of culturable species, accurate identification of each species present is difficult, very time-consuming, labor intensive and can be biased. The aforementioned isolation methods can be coupled with the following, more recently developed, protein- and DNA-based methods, to provide more robust identification.

### Protein-Based Methods

#### Enzyme Immunoassays

A sandwich Enzyme-Linked ImmunoSorbent Assay (ELISA) has been developed for the detection of whole cells of *B. cereus*, by recognizing surface antigens specifically associated with *B. cereus* cells. This assay was developed by multiple location immunization of animal models with whole cell immunogen to develop hybridomas and subtractive screen was used to eliminate cross reactivity with closely related species (Zhu L. et al., 2016). The subtractive screen ensured the mAbs are highly specific against *B. cereus* and the assay has a lower detection limit of 0.9 × 10^3^ cells/ml in phosphate buffered saline. This assay has been tested using food samples spiked with various pathogens without the need for culturing. It was highly effective at identifying *B. cereus* cells in mixed samples, without interference by the food matrix or influence by other related species. Although this ELISA for detection of surface antigens is specific for *B. cereus*, it is not clear if it can recognize spores as well as vegetative bacteria, or if it can distinguish between live and dead *B. cereus* (Zhu L. et al., 2016). Failure to detect spores could lead to a false negative result, whereas detection of free floating antigens from dead *B. cereus* cells could lead to false positive results. Additional culturing may be needed to detect cell numbers below the lower detection limit, and thus eliminate these concerns. Enzyme immunoassays have also been developed for the detection of *B. cereus* toxins (Wehrle et al., 2009; Cui et al., 2016). Specific conditions are needed to ensure efficient protein production. Casein hydrolysate-glucose-yeast with 1% glucose is used for the production of enterotoxins in *B. cereus*, and 10% skim milk medium is used for cereulide production in *B. cereus* (Cui et al., 2016). A negative result from a proteomic based assay would not imply that the bacteria is not present, rather protein synthesis might not be currently active.

#### Limitations of Protein Based Methods

The requirement for correct expression conditions in order to identify proteins of interest is a hugely limiting step in protein based method for species identification. This is particularly true for spore-forming bacteria, whose presence is of concern but are currently in a dormant state during sample testing. Such requirements for specific growth conditions increase the analysis time and complexity, which may not be possible for large scale analysis of many possible toxin producers in laboratory situations. Furthermore, it is expected that the proteinaceous nature of dairy samples would greatly impeded the sensitivity of any protein analysis performed without initial culturing, even if expression was occurring.

### DNA-Based Methods

#### Post-Culture DNA-Based Classification Methods

##### Random amplified polymorphic DNA polymerase chain reaction (RAPD-PCR)

Random amplified polymorphic DNA polymerase chain reaction uses short random primers to amplify multiple random DNA segments which, once visualized on an agarose gel, give unique patterns (Williams et al., 1990). Analysis of these fingerprints allows differentiation of species and strains by comparing profiles of various known strains (Ronimus et al., 1997, 2003). This method has been applied to colonies obtained from dairy powders in New Zealand to identify *Geobacillus stearothermophilus, Anoxybacillus flavithermus, Bacillus licheniformis*, and *B. subtilis* as the main contaminants of WMPs and SMPs, as well as buttermilk and goat milk powders (Ronimus et al., 2003). It has also been applied to whole and SMPs in Uruguay, correctly identifying the presence of *B. licheniformis, B. megaterium, B. pumilus, A. flavithermus*, and *B. subtilis* (Reginensi et al., 2011). Indeed, using this approach, *G. stearothermophilus, A. flavithermus*, and *B. licheniformis* have been identified as the dominant species in whole and SMPs from multiple countries including; Poland, Germany, Switzerland, France, Portugal, Netherlands, Great Britain, Ireland, Canada, USA, Mexico, Chile, Brazil, South Africa, Thailand, Australia, and New Zealand. *B. subtilis, B. circulans, Ureibacillus thermosphaericus, B. coagulans*, and *B. pumilus* have also being identified, albeit in lower quantities (Ruckert et al., 2004). A common feature of the RAPD-PCR approach is the highlighting of the 3–4 most dominant species. However, species of lower abundance might be the most interesting in terms of food security and spoilage. One exceptional study described the use of RAPD-PCR, and revealed a more in depth array of species, in Chinese dairy powders (**Table 1**) (Sadiq et al., 2016). Apart from identifying previously unreported species, other details worth noting are that *B. licheniformis, G. stearothermophilus*, and *A. flavithermus* were again established as being present in high abundance while, importantly, *B. cereus* group species were also identified. This observation obviously has implications for food safety (Sadiq et al., 2016). Although informative, analysis of the gel bands in RAPD PCR is very subjective allowing errors in classification and bias. Furthermore, the method requires time-consuming and laborious preparation of reference strains and there may also be variability between gels with the same samples, thus large-scale analysis would be difficult.

##### Sequencing housekeeping genes

Housekeeping genes are genes that are essential for the functions of the cell and viability of the organism, and thus typically contain highly conserved regions (Gil et al., 2004; Eisenberg and Levanon, 2013). Genes that contain such highly conserved regions at either end of a more variable region are particularly useful for strain identification purposes as the conserved regions can be targeted using degenerate primers to facilitate PCR amplification and sequencing of the variable region (Case et al., 2007). Identification of genera present is facilitated by comparison with databases of corresponding variable region sequences of known origin (Case et al., 2007). Many genes have been utilized for classification of species in fluid milk in the form of molecular typing (Durak et al., 2006). Other typing methods have been described for milk powder isolates of *Geobacillus* spp. and *B*. *licheniformis* based on variable number tandem repeat analysis (Seale et al., 2012; Dhakal et al., 2013). The 16S rRNA gene is ubiquitous among bacteria, and contains multiple conserved and variable regions making it extremely useful, in general, for taxonomic classification. However, 16S rRNA gene sequencing cannot differentiate between closely related species or subtypes and other housekeeping genes such as *gyrB* or *rpoB* have been utilized to do so (Durak et al., 2006; Case et al., 2007). Recently, both the *rpoB* and 16S rRNA genes have been used to characterize the contaminating psychrophilic, mesophilic, and thermophilic spore populations isolated from sweet whey, WPC, non-fat dry milk and acid whey powders. At least 14 different species were identified, with *B. licheniformis, Geobacillus* spp., and *Anoxybacillus* spp. being the most abundant (Miller et al., 2015). These methods have the potential to allow identification and monitoring of persistent species and subtypes throughout dairy powder processing plants (Seale et al., 2012; Dhakal et al., 2013). Although not currently employed in sequencing dairy powder isolates, *cpn60* (Durak et al., 2006; Schellenberg et al., 2011), *pycA, ccpA* (Liu et al., 2015), and *groEL* (Chang et al., 2003) have all been used to varying success in the sequencing of isolates from fluid milk (Durak et al., 2006), vaginal (Schellenberg et al., 2016) and marine (Liu et al., 2013) populations and remain as potential targets for future application to study dairy powder-associated microbes.

##### Pyroprinting

Pyroprinting utilizes sequencing by synthesis on multiple copy polymorphic loci simultaneously. The sequence reads are digitalized and can be compared using Pearsons correlation distance matrix to identify strains (Black et al., 2014). This method has been developed and utilized for source tracking, i.e., tracing sources of microbial contamination in end products or, more specifically, of endospore-forming bacilli in raw milk through to dairy powders. Presumptive species identified in powder included *G. thermoleovorans, A. flavithermus, B. licheniformis, B. pumilus*, and *B. amyloliquefaciens* (VanderKelen et al., 2016). These results correlate well with previous studies on raw milk and powders using the Sanger sequencing approach (Durak et al., 2006; Miller et al., 2015).

##### Limitations

All of the above tests allow identification of the most abundant culturable species identified in dairy powders. However, they are limited by an initial requirement for culturing and, unless these methods are modified for identification of species directly from dairy powders, they are not suitable for the identification of non-culturable species or species of lower abundance which can be out competed when culturing, unless selective media is employed. Ultimately, while promising, these methods when compared to culture-independent sequencing (see Next Generation Sequencing for the Identification of Dairy Powder Contaminants) are labor intensive and time consuming.

#### Targeted DNA Based Approach

A more targeted approach can be taken in the food sector to detect specific pathogens or groups of interest. These assays allow detection of toxin genes, possible pathogenic groups or members of a species of interest. Most of these have been adapted to allow amplification directly from mixed DNA extracted from foodstuffs and thus avoid the limiting step of culturing. Many also allow quantification of the species/toxin gene containing group. Of particular relevance to this review is the fact that a great deal of research has been performed with respect to such assays and the *B. cereus* sensu lato.

##### PCR assays

Polymerase Chain Reaction (PCR)-based assays have been developed for the detection of *B. cereus* toxin genes. Taqman quantitative PCR (qPCR) assay of a single component of the hemolysin toxin gene in *B. cereus* has been developed (Cattani et al., 2016), amplifying the sequence corresponding to one component of one tripartite toxin. It has been reported that the Taqman probe is specific for *B. cereus* strains that contain this gene, however, not all *B. cereus* strains contain the hemolysin gene (Cui et al., 2016). This assay reportedly does not give false positives with related species, such as other members of the *B. cereus* sensu lato including *B. thuringiensis* and *B. mycoides*. However, this assay could lead to false negatives. The assay may fail to detect other species that have the toxin genes, or other strains of *B. cereus* that do not have this particular toxin, but may be pathogenic due to the presence of other toxins. This assay also gives accurate quantification of viable *B. cereus* by comparison to standard curves. Multiplex endpoint PCR of toxin genes has also been performed to identify *B. cereus* in dairy samples. These assays included primers to amplify single components of *B. cereus* enterotoxin genes, i.e., those encoding Nhe, CytK, and Hbl (Zhang et al., 2016) as well as enterotoxin FM (EntFM) and emetic toxin Ces (Forghani et al., 2015). However, the specificity of these assays was only tested using *B. cereus* and non-*Bacillus* species. Multiplex PCR of multiple components of *B. cereus* toxin genes has also been performed on single bacterial colonies isolated from dairy products and environments (Wehrle et al., 2009). This approach allows detection of all components needed to produce viable enterotoxins, and thus lessening the chance of false readings compared to other assays that only identify one toxin gene component. Multiplex endpoint PCR assays have also been developed for hygiene indicator species, *G*. *stearothermophilus* and *A*. *flavithermus* isolated from dairy powders. These assays rely in the species specific conserved regions of ITS 16S-23S rRNA region and the rpoB gene (Pennacchia et al., 2014). Further validation of these assays could lead to their use on DNA isolated directly from dairy powders. Finally, droplet digital PCR (ddPCR) allows precise, absolute quantification of a target DNA sequence. The DNA is encapsulated into many water in oil emulsion droplets and a PCR performed on each (Pinheiro et al., 2012). This culture-independent method has recently been used to detect *B. cereus* in fluid milk and can provide absolute quantification without need for comparison to standard curves. In this instance ddPCR was implemented using primers that target the *gyrB* gene of *B. cereus* sensu lato and the assay was found to have a lower detection limit than traditional qPCR (Porcellato et al., 2016), which is ideal for dairy powders that have low levels of contamination.

##### Biosensors

The assays described above also have the potential to be employed in the form of biosensors. Indeed, biosensors are already being developed for detection of a toxin gene found in *B. cereus* in milk and powder (Izadi et al., 2016). These biosensors are DNA based pencil graphite electrode (PGE) biosensors, in which a *nhe* toxin gene primer is immobilized on gold nanoparticles. Positive results are measured by an increase in charge resistance on the biosensor from the hybridization of the target DNA to *nhe* toxin sequence.

##### Limitations of targeted DNA assays

Although these methods do not give a complete view of the microbial composition in a dairy powder, they are useful as a test for key spoilage and pathogenic bacteria, including producers of harmful toxins. It is important to note that *B. cereus* sensu lato toxin genes are not specific to any one species of the group, nor is one toxin found in all *B. cereus* (Liu et al., 2015; Cui et al., 2016; Zhu K. et al., 2016). However, targeting toxins allows detection of all possible pathogenic species. Singleplex assays that target one component of one toxin may be prone to false negatives (Cui et al., 2016), i.e., producers of other toxin types being overlooked, thus underestimating the number of pathogenic *B. cereus* cells in a sample. Multiplex assays targeting many toxins, are more robust and can be beneficial for the food industry as they are a good indicator of potential food pathogens. Targeting all components of a toxin system may be required to confirm if there is a true potential for toxin production. Furthermore, while the genes for toxins may be present, it is unclear from these assays whether any active proteins are functionally expressed. The alternative use of a non-toxin gene for identification of *B. cereus* (*gyrB*) does not distinguish between members of *B. cereus* sensu lato, nor does it identify if the species identified are capable of being pathogenic. Overall the detection of toxin and species specific genes are a good indicator of potential pathogenic and other species of interest being present. Although issues remain, future improvement and development should result in the full potential of these approaches being realized.

#### Culture-Independent, Non-targeted DNA Analysis

As outlined, there are limitations associated with the aforementioned culture-dependent and targeted assays. Culture-independent DNA-based analysis should be considered when striving to obtain an overview of all (i.e., culturable and non-culturable) spore-forming species present in dairy powders. This involves a shift away from testing for and identifying only specific known spore-forming bacteria in order to eliminate the possibility of currently unknown or underappreciated microbiology-related food security threats.

##### Next generation sequencing for the identification of dairy powder contaminants

In the last decade, considerable advances have meant that next generation DNA sequencing platforms have surpassed traditional Sanger sequencing platforms in terms of speed and potential applications. Their initially extremely short sequencing read lengths are less of a concern as sequencing lengths of Illumina and Ion platforms have increased (Quail et al., 2012) and new, even longer read, platforms have been developed by PacBio and Oxford Nanopore (Quail et al., 2012; Madoui et al., 2015). The advantages and disadvantages of the various sequencing platforms have been previously reviewed elsewhere (Goodwin et al., 2016). Regardless, research laboratories now have a much greater choice when determining which sequencing technology to use, though it should be noted that results generated using different methods, technologies or bioinformatics pipelines are not always consistent (Clooney et al., 2016). Whole genome shotgun sequencing is the process by where the whole genome of a single colony is sequenced. The DNA is extracted and sheared it into small pieces, before sequencing of these pieces and the use of computer software to assemble these sequences reads back together. This process can be applied to metagenomics, the term used to denote all of the genomic information from an entire community of different cells, for example the contaminants in dairy powders (Sharpton, 2014). The application of metagenomic techniques to the analysis of dairy products presents exciting opportunities. Metagenomic sequencing eliminates the need to culture, thus reducing bias, and allows the identification of species that are difficult to, or cannot be, cultured in the laboratory. Metagenomic sequencing has been applied to single gene products, such as the aforementioned 16S rRNA gene that can differentiate between all bacteria present to the genus level, while the *spo0A* gene has been targeted to specifically identify spore-forming Firmicutes in mixed populations. A whole metagenome ‘shotgun,’ i.e., untargeted, approach has also been attempted and comparison of 16S amplicon sequencing, *spo0A* amplicon sequencing and metagenomic shotgun sequencing performed for the identification of Firmicutes in metagenomic samples (Filippidou et al., 2015). Each method has advantages and disadvantages. Amplicon sequencing is more cost effective, high throughput and rapid but often only gives accurate classification to genus level, and may over-estimate microbial diversity in the sample (Acinas et al., 2004; Poretsky et al., 2014). In contrast, shotgun sequencing is more expensive, less samples can be analyzed at one time, but it gives the opportunity to accurately classify to species level provided there are accurate reference databases to compare sequence reads to Sharpton (2014). Shotgun sequencing also reduces the bias of amplicon sequencing that can arise due to need for an initial PCR amplification and, where relevant, variable gene copy numbers (Sharpton, 2014; Brooks et al., 2015). The other advantage of shotgun metagenomic approaches is that additional information regarding other genes of interest within the microbial community can be generated. Such genes include toxin genes (Steffen et al., 2012; Leonard et al., 2015), sporulation genes (Filippidou et al., 2015), non-ribosomal peptide synthase (NRPS) gene clusters (Schirmer et al., 2005), antibiotic resistance genes (Bengtsson-Palme et al., 2014), and phage genes (Dutilh et al., 2014), all of which may be interesting from a food safety point of view. The sequencing reads from this approach can be difficult to analyze as they can be biased toward genomes of higher abundance. This is a particular issue when studying samples from specific human and animal microbiomes where there is a considerable amount of DNA from host cells present (Feehery et al., 2013). It is important to note that, due to the high sensitivity of shotgun metagenomic sequencing, care needs to be taken to ensure the absence of contaminating cells or DNA from other environments (Salter et al., 2014; Glassing et al., 2016).

Regardless of the sequencing approach taken, bioinformatic expertise is needed to analyze sequencing data and compare sequence reads to databases. Databases and bioinformatics software are updated continuously and newer, more accessible programs are constantly being developed (Vincent and Charette, 2015), including more targeted programs and databases specifically for food microbes (Vangay et al., 2013; Parente et al., 2016).

##### Limitations

Both amplicon and shotgun metagenomic sequencing reveal the relative abundance of bacteria in a sample. Furthermore, the quantification of total bacterial load can be achieved by coupling these techniques with qPCR or ddPCR analysis, (Porcellato et al., 2016).

While the benefits of next generation sequencing in determining the safety and quality of dairy powders provide cause for optimism, there are several hurdles. Culture-independent DNA analyses rely on one’s ability to extract all genomic DNA directly from the substrate for analysis. Extracting DNA from dairy powder can be difficult, especially from spore-forming bacteria. Although, many studies have endeavored to optimize methods for the extraction of DNA from spores that have been spiked into food, success has been varied (Wielinga et al., 2011; Mertens et al., 2014). Furthermore, the bacterial load is likely to be lower in dried dairy powders than other environmental samples in which this sort of analysis has been previously performed, such as the gut (Gill et al., 2006), soil (Fierer et al., 2012), and fermented food (Jung et al., 2011). Low DNA concentration can be overcome through use of whole genome amplification kits (Yokouchi et al., 2006; Binga et al., 2008). Although expensive, these provide for culture independent non-targeted analysis of all bacteria present in dairy powders even if present at low cell numbers. However, these kits are notoriously susceptible to contamination (de Bourcy et al., 2014) and, ideally, ultra clean laboratory environments are needed for their use (Weinmaier et al., 2015).

*Isolation of DNA solely from spore-formers.* There may be instances where there is a specific desire to specifically focus on the sequencing of DNA from the spore-forming community within a powder sample. Isolation of DNA solely from spores/spore-forming bacteria is a challenge. One possible method would be to perform standard spore pasteurization at 80°C for 12 min (see Spore Count Methods) (Frank and Yousef, 2004; Watterson et al., 2014) or other forms of targeted vegetative cell lysis (Wunderlin et al., 2016). However, free DNA could still be present in the samples from the lysed vegetative cells. Elimination of this signal could be performed using an intercalating dye (described below). Post-heat treatment, subsequent culture-based enrichment could be employed prior to DNA extraction (Frank and Yousef, 2004; Watterson et al., 2014) but, as described with respect to the culture-based approaches, this has the potential to lead to bias.

Sequencing-based approaches can also be adapted to specifically focus on spore-formers by, for example targeting of the *spo0A* gene for amplicon sequencing, or through focusing specifically on this gene from within shotgun sequence data. However, yet again, the need to ensure optimal DNA extraction and the removal of DNA from dead cells is a key consideration. A less conventional way of overcoming such challenges could involve the isolation of spores from dairy powder using density gradient centrifugation (Tamir and Gilvarg, 1966).

As noted above, free DNA from lysed vegetative cells can be present in samples following heat-treatments. Elimination of this signal could be performed using an intercalating dye. The use of intercalating dyes is especially relevant in the case of amplicon metagenomic sequencing where PCR amplification is performed (Rudi et al., 2005). This has been performed utilizing the dyes propidium monoazide (PMA) or ethidium monoazide bromide (EMA) to bind free DNA in the samples (Rudi et al., 2005; Forghani et al., 2015; Cattani et al., 2016; Zhang et al., 2016). Further testing and optimization would be needed to determine if its results are as promising for dairy powder samples with mixed populations. There are contradicting studies with regard to whether EMA or PMA is best for particular applications (Seinige et al., 2014; Wu et al., 2015). Very few studies have compared EMA and PMA in mixed populations, though EMA was reported to be favorable at penetrating heat damaged bacterial cells in fish fillets (Lee and Levin, 2009). EMA has been known to penetrate some live bacteria (Nocker et al., 2006; Seinige et al., 2014) whereas PMA has been seen not to penetrate all dead cells (Cattani et al., 2016). The concentrations of EMA used has seen a decrease in recent years (possibly to circumvent the penetration of live cells) and, so, while early studies used 100 μg/ml (Rudi et al., 2005; Nocker and Camper, 2006), more recent studies used 8–10 μg/ml (Seinige et al., 2014; Wu et al., 2015). Alternatives, including the use of platinum (Soejima et al., 2016) to bind extracellular DNA, appear promising as they have been reported to be more selective at differentiating live/dead *E. coli* and *C*. *sakazakii* than PMA in water and milk. Ultimately, optimization needs to take place to develop the system that is best suited to the low microbial load of mixed populations present in powdered dairy products. It should also be noted that these approaches are not effective when performing metagenomic shotgun sequencing, as there is no amplification step to eliminate the dye-bound DNA.

##### Outlook

Currently culture-independent, population-based, analysis is relatively expensive and, thus, further developments are needed to increase its relevance to the food industry. It is, however, becoming more accessible as a test method for companies to strategically analyze processing pipelines and end products, allowing development of targeted treatments and intervention strategies against persistent or troublesome microorganisms. To provide thorough and reproducible analysis of dairy powders in this fashion, it will be particularly important to arrive at a consensus regarding the standardized sample preparation, use of specific sequencing platforms and analysis methodologies to facilitate comparison across multiple investigations (Clooney et al., 2016).

## Conclusion

Newer technologies have paved the way for an overhaul in the approaches taken to detect and enumerate of spore-forming bacteria in dairy powders. This can lead to a more accurate, high throughput system. Although the newer technologies themselves are not without their limitations, they are continuously improving. Optimization of these newer technologies could lead to their routine use, allowing development of improved targeted treatments and preventative measures in the powder processing industry.

## Author Contributions

AM drafted and edited the manuscript. Revised and edited by CF, CH, and PC.

## Conflict of Interest Statement

The authors declare that the research was conducted in the absence of any commercial or financial relationships that could be construed as a potential conflict of interest.

The reviewer HC and handling Editor declared their shared affiliation, and the handling Editor states that the process nevertheless met the standards of a fair and objective review.
